# Effect of chitosomes loaded zein on physicochemical, mechanical, microbial, and sensory characteristics of probiotic Kashk during cold storage

**DOI:** 10.1016/j.fochx.2024.101624

**Published:** 2024-07-04

**Authors:** Sara Karimi, Leila Nateghi, Elahesadat Hosseini, Mohammad Ali Fakheri

**Affiliations:** a Department of Food Science and Technology, Varamin-Pishva Branch, Islamic Azad University, Varamin, Iran; b Department of Food Science and Technology, National Nutrition Sciences and Food Technology Research Institute, Faculty of Nutrition Sciences and Food Technology, Shahid Beheshti University of Medical Sciences, Tehran, Iran; c Department of Chemical Engineering, Payame Noor University, Tehran, Iran; d Department of Food Science and Technology, Faculty of Pharmacy, Tehran Medical Sciences, Islamic Azad University, Tehran, Iran

**Keywords:** Chitosan, Functional Kashk, Nano-liposome, Probiotic, Zein

## Abstract

Functional foods like probiotics offer health benefits against various diseases, and plant bioactive compounds can enhance their growth. Zein, a protein, shows biological activity upon hydrolysis, and encapsulating it in nanoparticles improves bioavailability. This study examined chitosan-coated nanoliposomes as carriers for hydrolyzed and unhydrolyzed maize zein to fortify kashk. Combining chitosan and hydrolyzed zein in a 1:2 ratio achieves the highest encapsulation efficiency, antioxidant activity, smallest particle size, polydispersity index, and zeta potential. FTIR and XRD analyses confirm hydrolyzed zein's entrapment and crystalline nature post-encapsulation. Optimized nanoliposomes release hydrolyzed zein faster in simulated intestinal fluid than in gastric fluid, indicating high bioavailability and stability. When used to fortify kashk, these nanoliposomes slightly lower acidity but maintain standard pH over 60-day cold storage, improve elastic properties, and enhance probiotic viability. At the same time, sensory attributes remain comparable to the control, highlighting their functional food potential.

## Highlights

• Kashk, a traditional Iranian dairy product, is rich in protein.
• Chitosan-coated nanoliposomes with hydrolyzed and non-hydrolyzed zein were developed.
• Fortifying kashk with nanoliposomes loaded with hydrolyzed zein improves quality.
• Optimized nanoliposomes boost probiotic viability in kashk during storage.

## 1 Introduction

The recent shift towards consuming foods rich in essential nutrients that promote health underscores the critical role of digestive health in the development of functional food products. This trend is shaping the future of the food industry (Pourahmad & Hosseini, 2023). Probiotic products, among them kashk, are gaining attention in the field of functional foods. These microorganisms, due to their ease of production and ability to enhance the nutritional value and health benefits of products like yogurt, cheese, and fermented beverages, are playing a significant role in the development of functional food products (Keshavarzi et al., 2020; Sekhavatizadeh et al., 2019).

Kashk, a dairy product rich in protein and minerals, is a significant source of high-quality nutrition. It is commonly produced from salty byproducts like buttermilk or low-fat yogurt. (Hematian et al., 2020). Traditionally, kashk is obtained in dried form, while industrially, it is produced in liquid form. The dried variation necessitates soaking and softening prior to utilization. Specific standards, including a low pH (below 4.5), 20–25% nonfat solids, 1% fat, 3% salt, and a minimum of 13% protein, are endorsed by the Institute of Standard and Industrial Research of Iran for liquid kashk. Previous research examining samples from various provinces in Iran revealed that the protein content of dried kashk varied significantly, ranging between 39.6% and 67.8% (Shiroodi et al., 2012). Although kashk is rich in moisture and protein, it is highly susceptible to microbial contamination. Preservatives are not allowed in kashk; however, natural aromatic extracts or plants can enhance its flavor (Hematian et al., 2020).

Bioactive compounds such as carotenoids, ferulic acid, and anthocyanins from maize offer substantial health benefits, including antioxidant, anti-HIV, anti-inflammatory, and antimicrobial effects. They are essential for lowering the risk of chronic diseases like heart disease, type 2 diabetes, obesity, and certain cancers, and they also promote digestive health (Rouf Shah et al., 2016). Zein, an alcohol-soluble protein comprising 45–50% of maize endosperm protein, is considered GRAS (generally recognized as safe), non-toxic, and biodegradable. It forms spherical nanoparticles widely used in pharmaceuticals, food, and biotechnology (Liang et al., 2018; Mazloum-Ravasan et al., 2022). Despite being widely used, zein isolate (native zein) is unsuitable for direct human consumption due to its negative nitrogen balance and poor water solubility. Protein hydrolysis can be used to overcome these issues, which improves zein's functionality by chemically or biologically modifying its structure and properties (Mazloum-Ravasan et al., 2022; Najafi Rashed et al., 2024). Zein hydrolysate offers anti-inflammatory, antioxidant, and antibacterial benefits, but stomach acid might weaken these effects (Liang et al., 2018). Additionally, digestive enzymes break down the peptides. To effectively deliver these benefits, a carrier system is needed to protect them during their passage through the stomach and intestine (Li et al., 2015; Najafi Rashed et al., 2024).

In this regard, nanocarriers, particularly liposomes, due to their high surface area and biocompatibility (Pabast et al., 2018), offer promise for delivering zein hydrolysate through the digestive tract. Effective carriers should facilitate controlled release and improved bioavailability of the components while minimizing their toxicity (Choudhary et al., 2021; Pabast et al., 2018; Ramezanzade et al., 2017). Notably, liposomes are attractive due to their ability to shield bioactive compounds from stomach acid and enhance intestinal absorption, ultimately improving the bioactivity and bioavailability of encapsulated zein hydrolysate (Ramezanzade et al., 2017). Their unique structure allows them to encapsulate various molecules, including zein hydrolysate (Mazloum-Ravasan et al., 2022). However, liposomes face limitations, such as degradation by digestive enzymes and aggregation, which can hinder release.

Encapsulation within edible coatings like chitosan, a natural polysaccharide with mucoadhesive properties, can address these challenges. (Li et al., 2015; Pabast et al., 2018; Ramezanzade et al., 2017). Chitosan interacts with liposomes, enhancing their stability during processing and storage, protecting them from degradation, and preventing unwanted interactions with food components. (Guo et al., 2022). This synergistic approach using chitosan-coated liposomes holds promise for preserving the bioactivity of zein hydrolysate and achieving controlled release within the digestive tract (Rubaka et al., 2023).

This study investigated a novel delivery system for maize zein in probiotic kashk. Hence, chitosan-coated nanoliposomes were developed to encapsulate hydrolyzed and non-hydrolyzed zein at various ratios. Dynamic light scattering (DLS) assessed the size distribution, a critical physicochemical property of the nanoparticles. Encapsulation efficiency (EE) and antioxidant activity were determined using established assays (DPPH and ABTS). Optimized formulation underwent further characterization by FTIR and XRD to elucidate potential interactions between zein and the delivery system. Additionally, the gastrointestinal stability of the optimized formulation was evaluated. The focus shifted to the impact of these optimized nanoliposomes loaded with zein on probiotic kashk. Physicochemical, rheological, microbial, and sensory properties of the kashk were monitored during cold storage for 60 days, with e*v*aluations at specific intervals (0, 15, 30, 45, and 60 days). This investigation aimed to assess the potential of this delivery system to enhance the functionality and shelf life of probiotic kashk.

## 2 Material and method

### 2.1 Material

The commercial lyophilized cultures, including *Streptococcus thermophilus* and *Lactobacillus delbrueckii* ssp. *bulgaricus* were supplied by Chr. Hansen (Horsholm, Denmark). A probiotic culture comprising L. *acidophilus* LA-5 (Chr. Hansen, Horsholm, Denmark) was also used. Chitosan of medium molecular weight (MW: 190–310 kDa) with a deacetylation degree of 75–85% and zein from maize were obtained from Sigma-Aldrich Co., USA. Alkalase and papain enzymes were also sourced from Sigma-Aldrich Co. MRS agar, and *bile* salt contained *1*% and *2*% (*w*/*v*) *porcine bile extract* were acquired from Merck Co. (Germany) and Sigma–Aldrich (*Canada)*, respectively. All other chemicals necessary for the study were procured from Merck Co., ensuring analytical grade quality.

### 2.2 Preparation of protein hydrolysates

Zein hydrolysis was conducted in a two-stage process as outlined by Rezvankhah et al. (2021) with slight modifications. Alcalase and papain enzymes were utilized serially under their respective optimum conditions. The first hydrolysis stage commenced with Alcalase at an enzyme-to-substrate ratio (E:S) of 2% (*w*/w), a temperature of 60 °C, and a pH of 8. This stage lasted for 240 min. Afterward, the Alcalase enzyme was inactivated by heating the solution to 90 °C for 15 min; then, the solution was adjusted to the optimum conditions for papain (60 °C and pH 7.0). Papain was added at a concentration of 2% (w/w) E: S. The reaction proceeded for an additional 120 min using papain, resulting in a total sequential hydrolysis time of 360 min. After heating the solution to 95 °C, the enzyme was inactive for 10 min. After enzymatic hydrolysis, the mixture was centrifuged at 15000*g* for 15 min to separate the peptides from proteins. The resulting supernatant was then spray-dried using a DORSA tech apparatus, employing a drying air temperature of 160 °C (with an exhausting temperature of 80 °C) and an air flow rate of 0.3–0.4 MP. The resulting powdered hydrolysates, zein hydrolysate (ZH), were carefully placed in sterile sealed bags and kept at −18 °C until further use.

### 2.3 Preparation and chitosan coating of loaded nanoliposomes

Sarabandi et al. (2019) method was utilized for nanoliposome production. Initially, a solution comprising 0.09 g of phosphatidylcholine, 0.01 g of cholesterol, and 0.02 g of Tween-80 dissolved in 10 mL of absolute ethanol was stirred for 20 min. The solution was then moved to a flask and subjected to solvent evaporation and thin-layer formation at 60 °C with a rotational speed of 70 rpm using a rotary evaporator (Laborota 4002, Heidolph, Germany). Any remaining solvent residues were removed entirely after storing for 16 h in a desiccator. To create the liposomes, the thin layer was hydrated by adding 10 mL of hydrolyzed and non-hydrolyzed protein isolates (5 mg/mL) onto the film. The resultant solution was vortexed for 2 min and stirred in the rotary apparatus at 60 °C, with this process performed for 3 cycles. Particle size reduction was accomplished by subjecting the sample to 10 cycles of ultrasound treatment, with each cycle comprising 1 min of ultrasound followed by 1 min of rest. An ultrasound probe operating at a frequency of 20 kHz was employed for this purpose (UP200H, Hielsher, Germany). The nanoliposomes underwent coating following the method described by Li et al. (2015) with some adjustments. After initial experimentation, the nanoliposomes were combined with various concentrations (50, 100, and 200 mg/mL) of chitosan solution, prepared by dissolving chitosan in 1% (*w*/w) acetic acid at room temperature for 16 h. The mixture was then agitated for one hour at 300 rpm. The resulting coated samples, termed “chitosomes,” were stored at 4 °C until further analysis. Nanoliposome treatments containing different concentrations of chitosan and types of zein are given in Table 1.

**Table 1:** Ratio of chitosan /hydrolyzed and unhydrolyzed zein.

Treatment	Type of maize zein	Chitosan-Zein ratio(w/w)
T1	Non-hydrolyzed	2:1
T2	Non-hydrolyzed	1:1
T3	Non-hydrolyzed	1:2
T4	Hydrolyzed	2:1
T5	Hydrolyzed	1:1
T6	Hydrolyzed	1:2

### 2.4 Characterization of chitosomes

#### 2.4.1 Mean particle size, polydispersity index (PDI) and zeta potential

The physical characteristics of chitosomes, including mean diameter and PDI, were appraised utilizing DLS employing a Zetasizer instrument (NanoSiz Malven, Nano ZS 3000, UK). The nanoliposomes were diluted tenfold with phosphate-buffered saline to minimize the impact of multiple scattering phenomena. Subsequently, the diluted sample was introduced into a specialized cell designed for the DLS system, characterized by a width of 1 cm. Measurements were performed at room temperature and a scattering angle of 90°, employing a laser wavelength of 633 nm. Additionally, the zeta potential, which is an indicator of the nanoliposome's electrical charge, was determined using the same instrumentation. Measurements were conducted three times and reported as mean ± standard deviation.

#### 2.4.2 Assessment of Encapsulation Efficiency (EE)

Initially, 1 mL of the nanoliposomes prepared earlier was carefully relocated to an Amicon filter with a molecular weight cutoff of 10 kDa (Millipore, UK). Subsequently, the centrifugation process commenced at 3500 rpm for 10 min. This step aimed to assess the quantity of protein isolate not encapsulated within the liposomal matrix. The solution that permeated the filter was then utilized to ascertain the concentration of unencapsulated or free proteins, employing Bradford's method (Bradford, 1976). Finally, the EE, expressed as a percentage, was computed by dividing the amount of protein enclosed within the nanoliposomes by the initial protein concentration (Sarabandi et al., 2019).

#### 2.4.3 Antioxidant activity assessments

To assess the antioxidant activity of the chitosomes, each sample was subjected to a water bath at 100 °C for 5 min to ensure thorough release of the encapsulated compounds (Sarabandi et al., 2019). Following this, the antioxidant activity of the chitosomes was assessed using the procedures detailed in the subsequent sections.

##### 2.4.3.1 DPPH method

The DPPH radical scavenging activity of nanoliposomes was assessed. Briefly, 1.5 mL sample was combined with 1.5 mL of 0.2 mM DPPH dissolved in 95% ethanol. The mixture was vigorously shaken, left in the dark for 30 min, and then centrifuged for 10 min at 4000 ×*g*. The absorbance of the resulting solution was measured at 517 nm, and the DPPH radical inhibition was calculated using Eq. 1. The percentage of DPPH radical scavenging activity of nano-liposomal carrier treatments containing zein before and after hydrolyzation was calculated using the same formula.

(1) $$I\;\left(\%\right)=\frac{\mathrm{AC}-\mathrm{AA}}{\mathrm{AC}}\times 100\;$$

where AC represents the absorbance of the non-hydrolyzed sample, while AA represents the absorbance of the hydrolyzed sample.

##### 2.4.3.2 ABTS method

To perform the ABTS assay, a solution of ABTS radical cation (ABTS+) was generated by mixing 7 mM ABTS with 2.45 mM potassium persulfate. This mixture underwent incubation for 4 to 8 h at room temperature without light. The ABTS+ solution was then diluted with ethanol until it reached an absorbance of 0.7 ± 0.05 at 734 nm, as measured by an ultraviolet-visible spectrometer (WPA Lightwave S2000, Ireland). After this, 30 μL of each chitosome sample was mixed with 3 mL of the prepared ABTS+ solution, vigorously vortexed for 30 s, and left in the dark for 6 min. The inhibition of ABTS free radicals (%) was then computed using the same formula as described for the DPPH method.

### 2.5 Assessment of the characteristics of optimized chitosomes-loaded ZH

#### 2.5.1 FTIR analysis

Chitosan, zein, and the optimized chitosomes formulations containing ZH, selected based on the results acquired from the assays above, underwent FTIR spectroscopy analysis. The analysis was conducted using a Perkin–Elmer Model GX instrument based in the USA, employing the KBr technique at a ratio of 1:100. The spectral data of samples were acquired with a resolution of 4 cm^−1^, comprising 32 scans per sample and covering the wavenumber range of 4000–400 cm^−1^ (Najafi Rashed et al., 2024).

#### 2.5.2 XRD analysis

XRD pattern of compounds, including chitosan and zein, and the optimized chitosomes containing ZH were obtained through X-ray diffraction analysis (Bruker D8 Advanced, Germany). The diffraction pattern was obtained in the scattering angle (2θ) range between 5 and 40°.

### 2.6 Stability assay in gastrointestinal fluid

For evaluating the in vitro release pattern of the peptide from the optimized chitosomes-loaded ZH, we adopted a dialysis technique as described by Chang et al. (2017) with slight adjustments. Simulated gastric fluid (SGF) was prepared to mimic the gastric conditions during ingestion (pH 4.0), supplemented with 2 mg/mL of NaCl and 3.2 mg/mL of pepsin. Additionally, simulated intestinal fluid (SIF) with a final pH adjusted to 7.4 was obtained by mixing 8.8 mg/mL NaCl, 6.8 mg/mL KH_2_PO_4_, 2 mg/mL pancreatin, and 5 mg/mL bile salts.

For the test, the nanoliposome dispersion (3 mL) underwent dilution with SGF (3 mL) and then was positioned inside a dialysis membrane bag (molecular weight cutoff 14—8 kDa). The bag was submerged in 60 mL of SGF and incubated for 4 h. Subsequently, 6 mL of SIF was introduced into the dialysis bag, which was then relocated to a vessel containing 120 mL of SIF for an additional 2 h incubation period. These release procedures were conducted at a constant temperature of 37 °C with continuous agitation at 120 rpm. At designated time points in both environments, 1 mL of the release medium was sampled and replaced with an equal volume of fresh medium. The collected release medium was centrifuged for 10 min at 1000 ×*g*, and the supernatant was filtered through a 0.45 μm filter. The release of ZH from the nanoliposome into the medium was quantified using HPLC (Agilent, South Korea). A cumulative release profile of ZH over 6 h period was plotted using Eq. (2). This assay was conducted in triplicate.

(2) $$\mathrm{Cumulative release}\;\left(\%\right)=\frac{Q_{1}}{Q_{0}}\times 100\;$$

where Ql denotes the quantity of ZH released from the chitosomes at the time (t), whereas Qo signifies the initial amount of ZH contained within the chitosomes.

### 2.7 Liquid probiotic Kashk samples preparation

The probiotic kashk samples were prepared by the method outlined by Sekhavatizadeh et al. (2019) with slight modifications. Initially, milk fat was standardized to 3.5%. The standardized milk underwent homogenization at 100 to 200 kg/cm^2^ pressures, followed by pasteurization at 90 °C for 15 min. Subsequently, it was cooled to 43 °C. After adding the lyophilized starter culture and a single probiotic culture containing 10^9 CFU/mL of L. *acidophilus* (LA-5), the mixture was incubated at 43 °C until reaching optimal acidity (130°D). The resulting yogurt was refrigerated at 4 °C overnight. Next, it was relocated to a cooking tank heated to 80 °C for 4 h with constant stirring until it achieved a light brown color to form kashk. The total solids content (SNF) of the kashk was regulated to 20% *w*/w (SNF 14.28% *w*/w). Sterile salt at a concentration of 1% was introduced, and the sample was homogenized at 75 °C under pressures of 100–150 kg/cm^2^. Then, 1% (*w*/w) of the optimal ZH chitosome was added to the kashk. The prepared sample was placed in a sterile glass container, sealed, and refrigerated. Tests assessing physicochemical, microbial characteristics, rheological properties, and sensory attributes were conducted at 15-day intervals throughout the refrigerated storage period. The control sample comprised kashk without chitosome.

#### 2.7.1 Physicochemical properties of the Probiotic Kashk

The pH values of the kashk samples were determined using a pH meter (Greisinger Electronic, Germany). Subsequently, the percentage of lactic acid (according to the National Standard of Iran, 2852) was measured to indicate their titratable acidity. (Sekhavatizadeh et al., 2019).

#### 2.7.2 Rheological properties

Oscillation tests were performed using a rheometer (Anton Paar rheometer, model MCR300, Belgium) with parallel plate geometry. The gap and diameter between the plates were set at 0.02 mm and 0.05 mm, respectively. All tests were performed at a controlled temperature of 25 °C after homogenizing the samples. Subsequently, a certain amount of the samples was transferred between the parallel plates and allowed to equilibrate for 10 min to improve the sample structure. Silicon oil was applied to prevent moisture loss and solvent evaporation. Strain sweep test was first accomplished to determine the linear viscoelastic region under strains ranging from 0.1% to 100% with a frequency of 1 Hz to conduct oscillation tests. Then, the frequency sweep test (for measuring storage modulus (G') and loss modulus (G")) was performed in the linear viscoelastic region and a frequency range of 0.1 to 100 Hz with a constant strain of 0.5% (Mirarab Razi et al., 2018).

#### 2.7.3 Microbiological properties

Probiotic bacteria were selectively enumerated using MRS-bile agar. The plates were then incubated at 37 °C for three days under both aerobic and anaerobic conditions (Mortazavian et al., 2007). Probiotic cell populations were enumerated at 15-day intervals during the 60-day refrigerated storage period.

#### 2.7.4 Sensory evaluation

The sensory properties (texture, flavor, color, overall acceptability) were evaluated by a panel of 10 trained assessors aged between 25 and 40. They used a 5-point Hedonic scale (1: dislike extremely; 5: like exceptionally) to score each sample individually. The samples were provided to the panelists in individual plastic containers and were coded with three digits. The panel group randomly tested these codes. Between each test, water and biscuits were used to cleanse their palates and rinse their mouths (Sekhavatizadeh et al., 2019). All participants gave their informed consent to participate in the sensory study and to allow the use of their information in accordance with ethical research standards. Ethical permission was not required for this study because all the materials used were of food-grade quality, posing no risk to the participants.

### 2.8 Statistical analysis

In this study, 6 treatments of liposomal nanoparticles were prepared using a factorial design. The data were statistically analyzed using analysis of variance (ANOVA) and Duncan's test. SPSS software (version 32) was used for the analysis, and the graphs were plotted using Excel 2013.

## 3 Results and discussion

### 3.1 Physicochemical characterization of formulated chitosomes-loaded zein

Particle size and PDI, as fundamental physical properties of colloidal systems, profoundly impact stability, encapsulation efficiency, and bioactive compound release. The role of zeta potential in electrostatic interactions and the stability of encapsulation systems, as well as its impact on the binding and release of bioactive molecules from the carrier, has been evaluated via zeta potential analysis (Gharehbeglou et al., 2019). Our ANOVA analysis, as shown in Table 1S, revealed that the particle size spreading of loaded nanoliposomes was notably influenced by the ratio of chitosan to protein isolate, showing a more substantial effect than the type of protein isolates and their interaction (p ˂0.01). The implications of these findings are significant, as they provide a deeper understanding of the factors influencing nanoliposome properties. The impact of the chitosomes on the average size diameter, PDI, and zeta potential are summarized in Table 2. The average particle size data for the formulated chitosomes shows a range from 91.00 ± 1.00 nm for treatment No.6 to 188.00 ± 2.00 nm for treatment No.1. It's evident that particle size became more significant as the concentration of chitosan in nanoliposome increased (*p* < 0.05). The observed increase in particle size suggests a strong interaction between the nanoliposome and chitosan. This interaction might create a coating around the liposome, compromising its stability (Gharehbeglou et al., 2019). Furthermore, studies on bioactive ingredient encapsulation have demonstrated that excessive biopolymer use can promote interactions between biopolymer chains, leading to depletion flocculation, which is consistent with our findings (Emam-Djomeh & Rezvankhah, 2020; Pauluk et al., 2019).

**Table 2:** Mean particle size, PDI, Zeta potential, EE, DPPH, and ABTS values of non-hydrolyzed and hydrolyzed zein-loaded chitosomes.

Treatment	Average size (nm)	Polydispersity index (PDI)	Zeta potential (mV)	EE (%)	DPPH (%)	ABTS (%)
T1	188.00 ± 2.00^a^	0.412 ± 0.02^b^	−17.35 ± 0.01^a^	66.37 ± 0.21^f^	71.61 ± 0.37^f^	76.23 ± 0.53^f^
T2	133.00 ± 0.00^b^	0.442 ± 0.04^b^	−24.36 ± 0.01^b^	72.81 ± 0.32^d^	77.97 ± 0.51^d^	81.13 ± 1.00^d^
T3	96.00 ± 1.00^e^	0.392 ± 0.03^e^	−29.62 ± 0.03^b^	81.99 ± 0.03^b^	85.49 ± 0.54^b^	88.65 ± 0.70^b^
T4	138.00 ± 2.00 ^f^	0.434 ± 0.04^f^	−21.79 ± 0.04^e^	68.45 ± 0.21^e^	73.78 ± 0.76^e^	78.03 ± 0.52^e^
T5	101.00 ± 1.00^f^	0.430 ± 0.02^b^	−27.36 ± 0.04^d^	75.18 ± 0.31^c^	79.19 ± 0.54^c^	83.60 ± 0.36^c^
T6	91.00 ± 1.00^b^	0.388 ± 0.02^a^	−36.22 ± 0.01^f^	88.42 ± 0.40^a^	91.15 ± 0.41^a^	94.03 ± 0.35^a^

Values with similar letters in the column (Mean ± Standard Deviation) do not have a significant difference (*p* > 0.05). Polydispersity index (PDI), Encapsulation Efficiency (EE), 2,2-Diphenyl-1-picrylhydrazyl (DPPH), 2,2′-azino-bis (3-ethylbenzothiazoline-6-sulfonic acid (ABTS).

The PDI serves as a measure of dispersion uniformity, ranging from 0 to 1. Values above 0.5 suggest non-uniform particle distribution and potential aggregation. Therefore, low PDI values confirm the formation of uniformly distributed chitosan. As indicated in Table 2, the PDI index ranged from 0.388 ± 0.02 to 0.434 ± 0.04 across treatments, with treatment No. 6 showing the lowest PDI value of 0.388 ± 0.02. Consistently, our study observed PDI values below 0.45, indicative of representative homogeneous dispersion (Pauluk et al., 2019; Sarabandi et al., 2019). Moreover, the current study revealed a strong link between the zeta potential of loaded nanoliposomes and the stability of the nanoparticle suspension. Significant variations (*p* < 0.05) were observed in zeta potential values across the samples, indicating substantial influence from chitosan levels. Treatment 1, containing the highest chitosan to hydrolyzed zein ratio (2:1), exhibited the least negative zeta potential (17.35 ± 0.01 mV). Conversely, treatment 6, with the lowest ratio (1:2), displayed the most negative value (36.22 ± 0.01 mV) (Table 2**).** Higher chitosan concentrations led to a less negative surface charge on the nanoliposomes, promoting favorable interactions between them and chitosan due to the presence of amino groups in chitosan. Furthermore, a correlation was found between zeta potential and suspension stability. (Loureiro et al., 2022; Seyedabadi et al., 2021). Zeta potential values exceeding ±30 mV indicated enhanced stability due to stronger repulsive forces between particles, suggesting effective chitosan coating that minimized aggregation (Pauluk et al., 2019).

Encapsulation efficiency (EE), a measure of a liposome's ability to retain its contents, is crucial for assessing the physical stability of encapsulated compounds (Reyhani Poul & Yeganeh, 2022). It also influences other properties like oxidative stability, morphology, and release rate (Sarabandi et al., 2019). The ANOVA analysis demonstrated that the concentration (chitosan to zein ratio) had a greater impact on EE than the zein isolate type (Table 1S). Table 2 shows the EE% of chitosan-coated zein nanoliposomes, ranging from 66.37 ± 0.21% to 88.42 ± 0.40%. Treatment 1 (chitosan: non-hydrolyzed zein, 2:1) had the lowest EE (66.37 ± 0.21%), while Treatment 6 (chitosan: hydrolyzed zein, 1:2) displayed the highest (88.42 ± 0.40%). The increased EE is primarily attributed to the chitosan coating layer, which hinders the release of loaded compounds. Chitosan molecules adhere to the liposome surface, forming a rigid shell that restricts lipid movement and increases resistance (Seyedabadi et al., 2021). However, doubling the quantity of chitosan led to a notable reduction in this index (Ramezanzade et al., 2017). Our findings are in agreement with previous studies by (Li et al., 2015; Ramezanzade et al., 2017; Sarabandi & Jafari, 2020), who investigated the impact of chitosan concentration on the EE of nanoliposomes loaded with various biomaterials. These studies collectively demonstrate that chitosan concentration is crucial in optimizing this process.

### 3.2 Antioxidant activity of chitosomes-loaded Zein

This study also investigated the influence of chitosan and zein protein isolate (hydrolyzed and non-hydrolyzed) on the antioxidant activity of chitosomes using DPPH and ABTS assays. ANOVA analysis revealed that the chitosan-to-zein ratio had the most significant impact on antioxidant activity compared to the type of zein isolate (hydrolyzed vs non-hydrolyzed) and their interaction (Table 1S). Increasing zein content, particularly hydrolyzed zein, significantly enhanced antioxidant activity (*p* < 0.05). The free radical scavenging activity of the bioactive peptides, as measured by DPPH and ABTS assays, ranged from 71.61 ± 0.37% to 91.15 ± 0.41% and 76.23 ± 0.53% to 94.03 ± 0.35%, respectively. Interestingly, treatment No. 6 (chitosan to hydrolyzed zein ratio of 1:2) exhibited the most potent inhibition against both DPPH and ABTS radicals compared to other formulations (p < 0.05). This enhanced activity might be due to two factors. Firstly, aromatic amino acids like leucine, proline, and histidine in the zein peptides likely facilitate proton donation to free radicals, effectively neutralizing them (Pauluk et al., 2019; Sarabandi & Jafari, 2020). Secondly, various interaction mechanisms between the chitosomes and free radicals could play a role. These mechanisms include intra-membrane transfers, contact release, binding and attachment, and absorption. (Reyhani Poul & Yeganeh, 2022). Additionally, chitosan itself possesses antioxidant properties, potentially contributing to the nanoliposomes' overall free radical scavenging activity. (Kulawik et al., 2023).

### 3.3 Characteristics of optimized chitosomes-loaded ZH

According to the findings of this study, the combination of chitosan and hydrolyzed zein in a 1:2 ratio resulted in chitosomes with the highest EE, the lowest mean particle size, PDI, and zeta potential, and the most potent antioxidant activity. These characteristics suggest that treatment No. 6 effectively protects bioactive compounds from degradation and offers a cost-effective solution compared to directly using these compounds in functional foods.

#### 3.3.1 FTIR

**Fig. 1:**
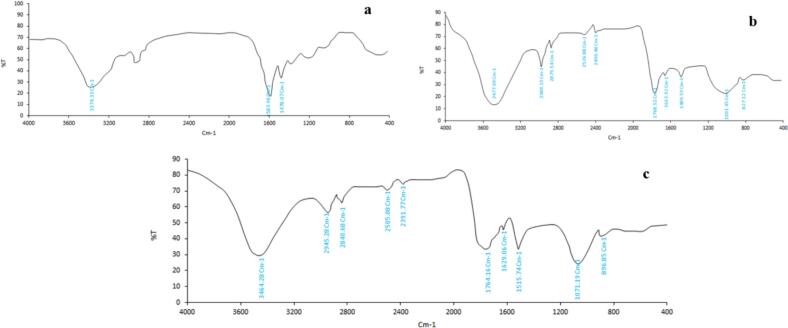
FTIR analysis of a) zein, b) chitosan, and c) optimized chitosome-loaded ZH.

The FTIR analysis aimed to identify the chemical bonds and structures within the nanoparticles and their coating materials. FTIR spectra of native zein, chitosan, and optimized chitosome-loaded ZH (1:2) are depicted in Fig. 1. Native zein exhibits distinctive peaks at 3299, 2931, and 2945 cm^−1^, corresponding to the stretching vibrations of (−NH_2_) and (−CH) asymmetric and symmetric bonds, respectively. These bands may potentially overlap with hydrogen bonds or N—H group vibrations. Moreover, amide vibrational bands at 1583 cm^−1^ (amide I) and 1478 cm^−1^ (amide II) were detected, with amide I attributed mainly to carbonyl C=O stretching vibrations, while amide II comprises both C—N stretching and C-N-H in-plane bending.(Najafi Rashed et al., 2024). Signals ranging from 800 to 1200 cm^−1^ originate from polysaccharides in the gluten, slightly affecting the analysis. Characteristic peaks of chitosan display a wide-ranging, intense band at 3477 cm^−1^ ascribed to O—H vibrational stretching (Fig. 1b). Additionally, peaks for chitosan were observed at 2980 cm^−1^, 2875 cm^−1^, 2525 cm^−1^, and 2400 cm^−1^, as well as at 1769 cm^−1^, 1663 cm^−1^, and 1489 cm^−1^ indicative of amide I and amide II, respectively. The FTIR spectra of chitosan-loaded zein nanoliposomes reveal bands of N—H stretching vibrations at 3464 cm^−1^, C—H stretching vibrations at 2945 and 2840 cm^−1^, and amide I and amide II bands at 1629 cm^−1^ and 1515 cm^−1^, respectively (Fig. 1c). Notably, upon the production of nanoliposome, a noticeable change occurred in the absorption peak related to O—H, the amide I, and amide II groups. Compared to zein and chitosan, nanoliposome absorption peaks were shifted slightly to 3464 cm^−1^, 1629 cm^−1^, and 1515 cm^−1^, respectively. This shift may be attributed to the formation of hydrogen bonds and cross-linking between the amide groups of zein and the hydroxyl groups of chitosan via electrostatic interactions (Sarabandi & Jafari, 2020). The investigation revealed analogous outcomes for zein-rhamnolipid nanoparticles enclosing curcumin. The interplay among zein, curcumin, and rhamnolipid via electrostatic forces led to a discernible absorption peak for amide II, contrasting with that of native zein and rhamnolipid (Dai et al., 2018).

#### 3.3.2 XRD

**Fig. 2:**
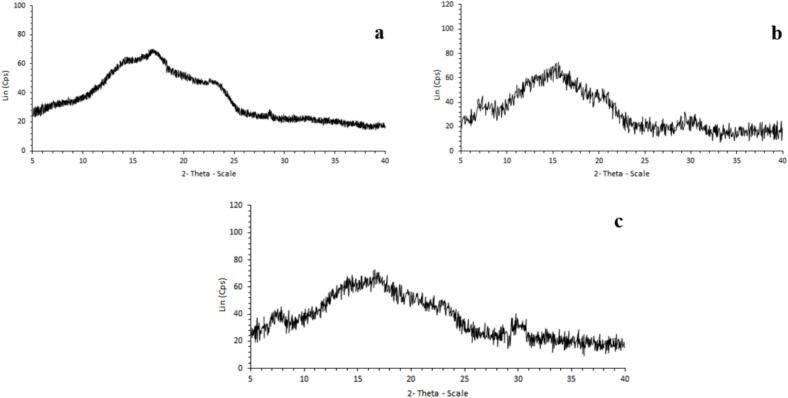
XRD analysis of a) zein, b) chitosan, and c) optimized chitosome-loaded ZH.

XRD diagrams compare X-ray energy values with standard element values for qualitative identification of elements in samples; while also enabling percentage determination. Peaks in XRD diagrams serve as indicators for identifying specific substances. Fig. 2a–c illustrates the crystal diffraction data and crystal state of biopolymer matrices, including hydrolyzed zein, chitosan, and chitosome-loaded ZH. All samples exhibited distinct peaks ranging from 5 to 30°, with smaller peaks extending to 40° 2θ. However, in the case of zein, closely spaced and compressed peaks were observed compared to the other samples, indicating a decrease in crystallinity. As shown, zein exhibited broad peaks at 17° and 23.5° at the angle 2θ, which means the crystalline nature of the native amorphous protein (Najafi Rashed et al., 2024; Wei et al., 2019). However, the deflection peaks of the chitosan showed peaks at 2θ =7°, 16°, 21°, and 30°, which were characteristics of chitosan (Yahya et al., 2006). The X-ray diffractogram of the optimized chitosan-coated nanoliposome (Fig. 2c) showed a broad peak at 2θ =7°, 17°, and 30°. The peaks at 2θ =23.5°, which were seen in zein, have disappeared, and the peak at 2θ =30° was intensified due to the interactions between zein and chitosan, confirming the embedment of ZH in the chitosome. The diffractogram suggested that the chitosome-loaded ZH was crystalline and had slightly altered the chitosan structure. Khan et al. (2019) reported similar findings, noting that the distinct peaks associated with zein, alginate, chitosan, and resveratrol were not observable in the XRD pattern obtained from zein–alginate–chitosan nanoparticles encapsulating resveratrol.

#### 3.3.3 Evaluation of ZH release from optimized Chitosomes through in vitro assessment

**Fig. 3:**
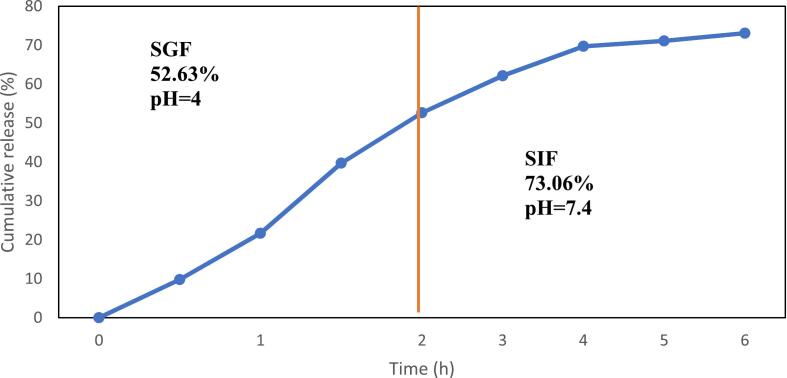
In vitro release profile of the optimum chitosomes-loaded ZH in simulated gastric fluid (SGF) and simulated intestinal fluid (SIF).

The gastric emptying time for a meal is typically around 112 min, while the average transit time in the small intestine ranges from 2 to 4 h (Cann et al., 1983). Therefore, the release of peptides in SGF has been investigated over a period of 2 h, and in SIF for a period of 4 h to mimic intestinal conditions. According to the results presented in Fig. 3, it is evident that the interaction between chitosomes and the simulated conditions, facilitating the release of ZH, was statistically substantial (*p* < 0.05). In chitosome nanocarriers, the release rate in SGF was more pronounced than SIF's. Furthermore, 52.36% of ZH was released from chitosomes as the duration extended from 0 to 2 h in SGF, while the release trend in SIF mirrored that of SGF, with a gradual release from 2 to 6 h, resulting in 73.06% of the total ZH being released in SIF. Hence, it can be deduced that the remaining ZH may be released over a longer duration in SIF. The observed phenomenon can be attributed to various factors. These include the acidic pH of SGF, which reduces the surface charge of complexes. Additionally, the presence of ionic compounds in SGF induces an electrostatic screening effect, facilitating the attraction of ions with opposite charges and thereby reducing the electrostatic repulsion between nanoliposomes. Furthermore, the complex structures were likely disrupted due to enzymatic hydrolysis (Emam-Djomeh & Rezvankhah, 2020). Reports suggest that when nanoparticles come into contact with gastric and intestinal fluids, gastric fluid's acidic and ionic conditions initially cause swelling and weaken the electrostatic interactions of composite layers formed by biopolymers. This process results in the partial release of bioactive compounds. Upon entering intestinal conditions, the enzymes and highly alkaline environment further disintegrate the biopolymer complexes, leading to the maximum release of entrapped bioactive compounds (Rezvankhah et al., 2020; Sarabandi et al., 2019; Seyedabadi et al., 2021).

### 3.4 Effect of addition of optimized chitosomes-loaded ZH on quality properties of Probiotic Kashk

#### 3.4.1 Physicochemical properties

##### 3.4.1.1 Acidity and pH

Changes in the acidity and pH of the fortified kashk sample with chitosomes-loaded ZH and the control sample during 60 days of storage at 4 °C are presented in Table 3. Initially, on the first day of storage, the acidity of the control samples and fortified kashk samples measured 1.399% ± 0.014% and 1.394% ± 0.012%, respectively, which later increased to 1.409% ± 0.04% and 1.404% ± 0.009% at the end of the storage time. No substantial disparity was detected in the acidity levels between the control and fortified kashk groups. However, the slight increase in acidity observed in the fortified samples is likely due to post-acidification during storage, a phenomenon caused by the ongoing activity of lactic acid bacteria. (Zhang et al., 2021). Similar findings were reported by Goyal et al. (2016) in their study on the fortification of curd with encapsulated flaxseed oil, which no significant change in acidity was observed. Additionally, Choudhary et al. (2021) reported similar results for fortified curd with chia oil nanoliposome.

**Table 3:** Acidity, pH, and Probiotic bacteria of control and optimized chitosomes-loaded ZH-fortified kashk.

	Acidity (Lactic acid %)		pH		Probiotic Bactria (Log CFU/mL)	
Day	Kashk (Control)	Kashk (Chitosomes)	Kashk (Control)	Kashk (Chitosomes)	Kashk (Control)	Kashk (Chitosomes)
0	1.399 ± 0.014^Aa^	1.394 ± 0.012 ^Aa^	4.47 ± 0.020^bA^	4.51 ± 0.01^aA^	8.814 ± 0.105^Aa^	8.80 ± 0.187^Aa^
15	1.402 ± 0.01 ^Aa^	1.397 ± 0.01 ^Aa^	4.44 ± 0.010^bB^	4.48 ± 0.01^aB^	7.735 ± 0.142^Ba^	7.877 ± 0.09^Ba^
30	1.045 ± 0.014 ^Aa^	1.398 ± 0.013 ^Aa^	4.43 ± 0.010^bB^	4.47 ± 0.02^aB^	7.371 ± 0.123^Ca^	7.493 ± 0.078^Ca^
45	1.406 ± 0.011 ^Aa^	1.401 ± 0.008 ^Aa^	4.40 ± 0.020^bC^	4.45 ± 0.01^aBC^	6.584 ± 0.116^Da^	7.182 ± 0.163^Ea^
60	1.409 ± 0.04 ^Aa^	1.404 ± 0.009 ^Aa^	4.39 ± 0.010^bC^	4.44 ± 0.02^aC^	5.759 ± 0.102^Fa^	6.772 ± 0.180^Ga^

Different lowercase letters in each column represent significant differences during the time(p ≤ 0.05). Different capital letters indicate significant differences among probiotic kashk samples simultaneously (*p* ≤ 0.05). Data (mean ± standard deviation) are from three replications.

In contrast to the control group, the pH values in the kashk fortified with chitosomes significantly increased (*p* < 0.05) during storage. Specifically, the pH of the fortified groups started at 4.51 ± 0.01 and reached 4.44 ± 0.02 by day 60, while the control groups decreased to 4.39 ± 0.010. Despite the decrease, the pH values remained within an acceptable range (4.39 to 4.51). These results align with the findings of Choudhary et al. (2021) who reported that fortification of crud with chia oil nanoliposomes led to a significant increase in pH.

#### 3.4.2 Rheometery results

**Fig. 4:**
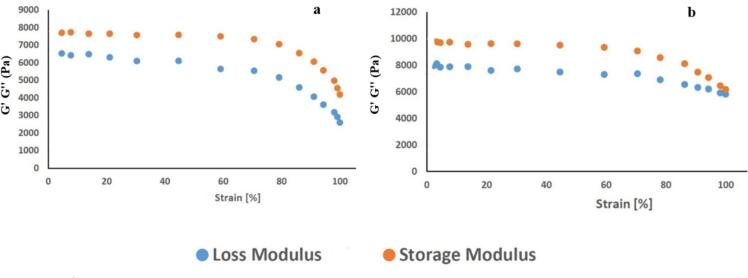
G′ and G″ versus strain for the control samples (a) and sample containing optimal chitosomes-loaded ZH (b).

The strain sweep test conducted at a fixed angular frequency of 1 Hz provided insights into the linear viscoelastic behavior of the kashk samples, aiding in determining the appropriate strain for subsequent frequency sweep tests. The results, depicted in Fig. 4 (4a and 4b), revealed two distinct regions: linear viscoelastic and nonlinear viscoelastic.

**Fig. 5:**
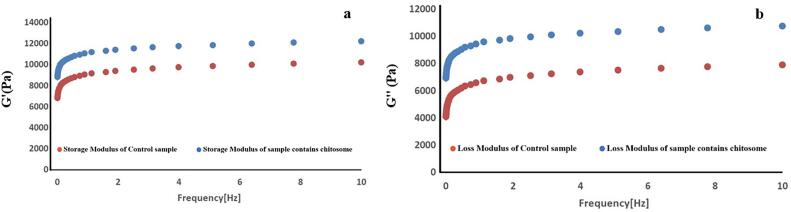
Storage modulus (G') (a) and loss modulus (G") (b) versus frequency for the control samples and sample-optimized chitosomes-loaded ZH.

Within the linear viscoelastic region, both the storage modulus (G') and loss modulus (G") began to change after remaining relatively constant, eventually showing a reduction. Notably, kashk samples containing chitosome exhibited higher values of G' and G" the control samples, indicating an enhancement in elastic properties due to the presence of nanoliposomes. Additionally, the G' remained higher than the G" over a wide range of strains in both samples, indicative of solid-like viscoelastic behavior. (Mezger, 2006; Shiroodi et al., 2012). Based on these results, an appropriate strain within the linear viscoelastic region can be determined for subsequent frequency sweep tests. Ensuring that the material response remains within the linear viscoelastic regime allows for accurately characterizing its viscoelastic properties. Fig. 5 (5a and 5b) shows the variations of frequency sweep tests in the linear viscoelastic region of kashk samples. These tests were performed at a fixed strain of 0.5% based on the results of strain sweep tests.

The figures exhibit a frequency-independent pattern, indicating a strong and regular structure within the samples. As frequency increases, resulting in a shorter observation time for deformation, smaller and more mobile molecules become less flexible, leading to an upward slope in the G' and G" curves (Shiroodi et al., 2012). Moreover, within the tested frequency range, the G' remains higher than the G", affirming the solid-like behavior of the kashk samples (Shiroodi et al., 2012). The absence of a crossover point further confirms this behavior, indicating strong and cohesive mechanical properties. Furthermore, both G' and G" in the sample containing chitosomes are higher compared to the control samples, suggesting a positive impact on the system's rheology. This increase in elastic modulus (G') signifies an enhancement in the mechanical and rheological properties of the nano liposome-containing ZH compared to the control.

#### 3.4.3 Microbiological quality

According to the results from Table 3, the influence of nanoliposomes on probiotic survival was negligible (*p* > 0.05), while storage duration had a significant impact (*p* ≤ 0.05). Chitosomes containing ZH effectively preserved probiotic viability throughout storage. *Lactobacillus acidophilus* LA-5 counts remained higher in chitosomes-loaded ZH compared to the control groups at all time points. After 60 days of storage, the probiotic viability in nanoliposome treatments reached 6.772 ± 0.180 CFU/mL, significantly higher than the control groups (5.759 ± 0.102 CFU/mL). This enhanced viability could be attributed to the protective properties of liposomes. They may shield the encapsulated peptides from degradation by bacterial enzymes, ultimately promoting ingredient bioavailability. These findings support previous research by (Salama et al., 2020), who demonstrated that fortifying yogurt with liposomes containing specific probiotic strains improved their viability during storage. Additionally, all probiotic samples containing chitosomes on the last day of storage contained probiotic bacteria at levels exceeding 10^6^. Generally, the food industry aims for bacterial populations exceeding 10^6^ probiotics/g at the time of consumption of the strain added to food (Ghorbanzade et al., 2017).

#### 3.4.4 Sensory properties

**Fig. 6:**
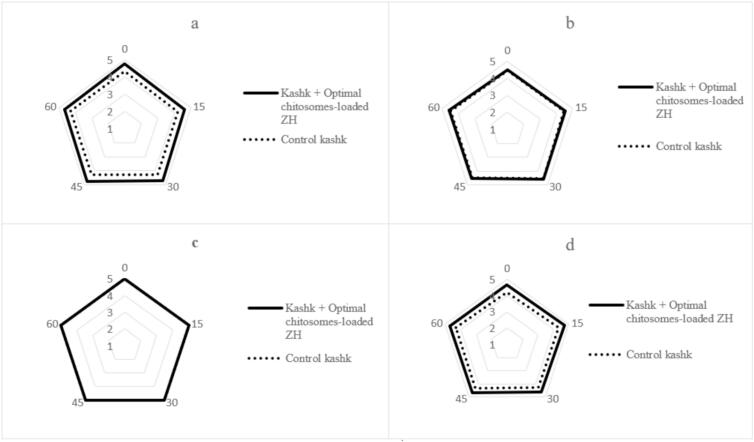
Sensory parameters of control and optimized chitosomes-loaded ZH -fortified kashk samples during storage for 60 days, Consistency(a), Flavor(b), Color(c), Overall acceptability(d).

To assess the impact of optimal chitosomes-loaded ZH in probiotic kashk, sensory evaluation was conducted on organoleptic properties compared to the control over a 60-day storage period. Fig. 6 illustrates the sensory scores of the kashk samples. The sensory analysis revealed no vital difference (p > 0.05) in flavor and color between both kashk samples during the storage (Fig. 6 b and c). However, there was a notable increase in scores for consistency (Fig. 6 a) in the presence of chitosomes-loaded ZH, indicating an enhancement in product consistency (p ≤ 0.05). Notably, kashk containing chitosomes demonstrated a significant improvement in overall acceptance (Fig. 6 d) (p ≤ 0.05). The findings align with those of Ghorbanzade et al. (2017) who observed no substantial effects on sensory parameters when fish oil encapsulated in nanoliposomes was incorporated into yogurt. This suggests that the addition of nano-encapsulated fish oil to yogurt yields sensory scores comparable to those of the control sample.

## 4 Conclusion

This study successfully encapsulated ZH, a form of zein broken down into smaller peptides, within chitosomes, and compared to unhydrolyzed zein, the physicochemical and antioxidant properties of the encapsulated ZH exhibited significant improvements. The optimal formulation was achieved with a 1:2 ratio of hydrolyzed zein to chitosan. FTIR and XRD analyses confirmed the successful encapsulation of ZH within the chitosomes. In vitro release studies demonstrated that the chitosomes offered the controlled release of the encapsulated ZH under simulated digestive conditions. This suggests promising potential for targeted delivery of ZH.

The optimal formulation's addition to probiotic kashk yielded promising results. While the acidity and pH remained almost unaffected, the rheological properties improved, and probiotic viability was enhanced during storage. Sensory evaluation indicated consumer acceptance of the fortified kashk. This study demonstrates the effectiveness of chitosomes-loaded ZH as a delivery system for bioactive compounds. These findings hold significant promise for functional food and nutraceutical applications. Further research and optimization are warranted to explore their full potential in various food matrices and therapeutic applications.

## CRediT authorship contribution statement

**Sara Karimi:** Formal analysis. **Leila Nateghi:** Supervision, Formal analysis, Data curation. **Elahesadat Hosseini:** Writing – review & editing, Writing – original draft, Validation, Investigation, Formal analysis. **Mohammad Ali Fakheri:** Formal analysis.

## Declaration of competing interest

The authors declare that they have no known competing financial interests or personal relationships that could have appeared to influence the work reported in this paper.

## Data Availability

Data will be made available on request.

## Appendix A Supplementary data

Table 1S: ANOVA for the characteristics of chitosomes-loaded zein. Image 1
