# Supplementary material for: Effect of chitosomes loaded zein on physicochemical, mechanical, microbial, and sensory characteristics of probiotic Kashk during cold storage
Source: Food Chem X. 2024 Jul 4;23:101624. doi: 10.1016/j.fochx.2024.101624 (PMC11295914; doi:10.1016/j.fochx.2024.101624)

**Supplementary Material**

Table 1S: ANOVA for the characteristics of chitosomes-loaded zein

| Source of Variation | Encapsulation Efficiency | | Particle size | | Zeta potential | | ABTS | | DPPH | |
| --- | --- | --- | --- | --- | --- | --- | --- | --- | --- | --- |
|  | F-value | p-value | F-value | p-value | F-value | p-value | F-value | p-value | F-value | p-value |
| Chitosan/Zein Ratio (A)) | 6243.695 | 0.000^**^ | 5623.875 | 0.000^**^ | 200079.338 | 0.000^**^ | 797.992 | 0.000^**^ | 1282.566 | 0.000^**^ |
| Type of Zein (B) | 762.795 | 0.000^**^ | 2838.375 | 0.000^**^ | 158723.787 | 0.000^**^ | 120.452 | 0.000^**^ | 140.414 | 0.000^**^ |
| Interaction Effect (A☓B) | 113.832 | 0.000^**^ | 577.125 | 0.000^**^ | 769.570 | 0.000^**^ | 13.855 | 0.001^**^ | 28.050 | 0.001^**^ |

****Indicates significance at the 1% level (p < 0.01)**


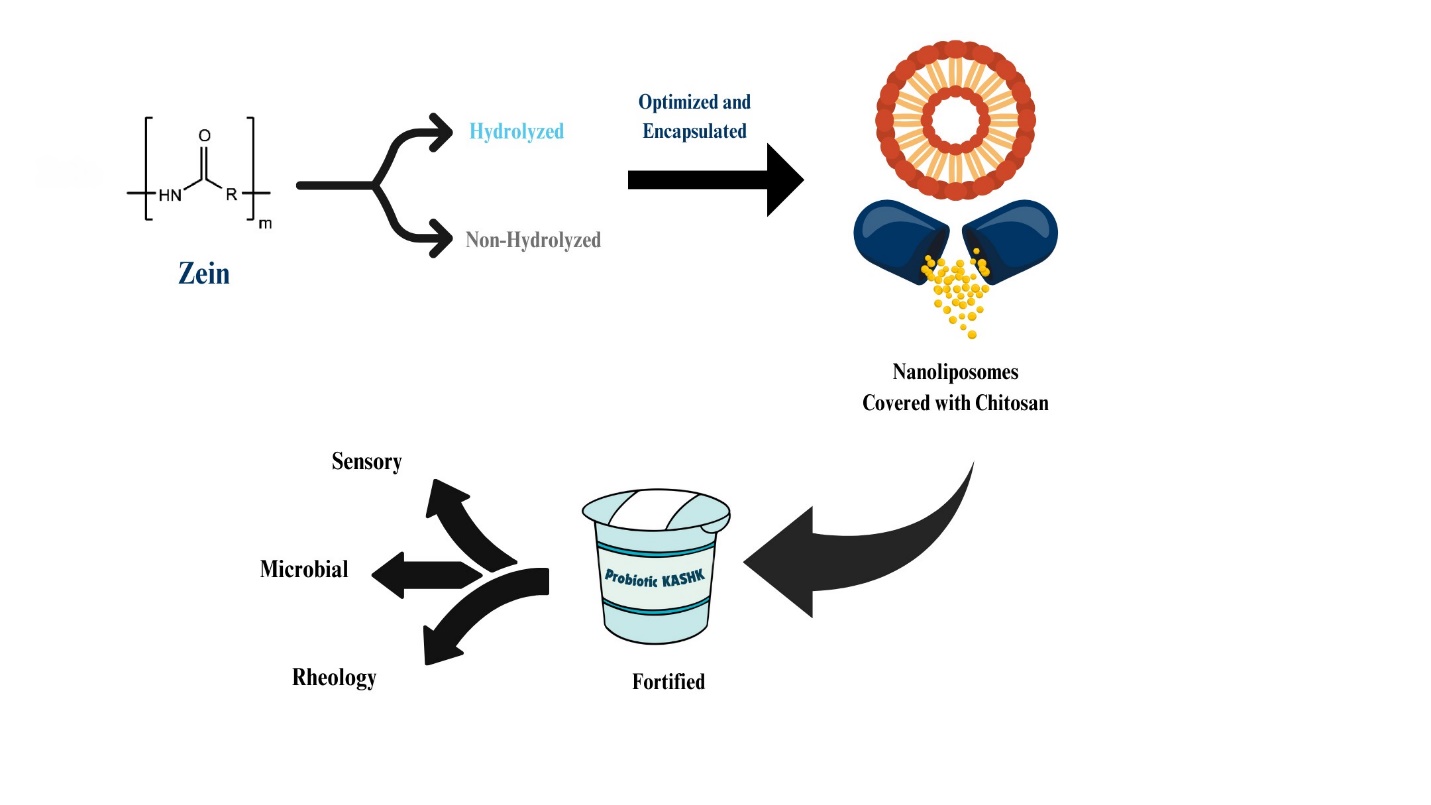

Supplement: Supplementary file 1 — Table 1S: ANOVA for the characteristics of chitosomes-loaded zein. [file mmc1.docx]
